# Value of oral glucose tolerance test in the acute phase of myocardial infarction

**DOI:** 10.1186/1475-2840-10-21

**Published:** 2011-03-11

**Authors:** Agata Bronisz, Marek Kozinski, Przemyslaw Magielski, Tomasz Fabiszak, Joanna Gierach, Iwona Swiatkiewicz, Adam Sukiennik, Aldona Kubica, Marek Bronisz, Zofia Grabczewska, Anna Sinkiewicz, Roman Junik, Jacek Kubica

**Affiliations:** 1 Department of Endocrinology and Diabetology, Nicolaus Copernicus University, Ludwik Rydygier Collegium Medicum, Bydgoszcz, Poland; 2 Department of Cardiology and Internal Medicine, Nicolaus Copernicus University, Ludwik Rydygier Collegium Medicum, Bydgoszcz, Poland; 3 Department of Health Promotion, Nicolaus Copernicus University, Ludwik Rydygier Collegium Medicum, Bydgoszcz, Poland; 4 Department of Cardiology, PS ZOZ Regional Hospital, Inowrocław, Poland; 5 Department of Phoniatrics and Voice Rehabilitation, Nicolaus Copernicus University, Ludwik Rydygier Collegium Medicum, Bydgoszcz, Poland

## Abstract

**Background:**

Although European guidelines advise oral glucose tolerance test (OGTT) in patients with acute myocardial infarction (AMI) before or shortly after hospital discharge, data supporting this recommendation are inconclusive. We aimed to analyze whether disturbances in glucose metabolism diagnosed before hospital discharge in AMI patients represents a latent pre-existing condition or rather temporary finding. Additionally, we planned to investigate the value of pre-selected glycemic control parameters as predictors of long-term glucometabolic state.

**Methods:**

We assessed admission glycemia, glycated hemoglobin, mean blood glucose concentration on days 1 and 2 in 200 patients with a first AMI but without overt disturbances of glucose metabolism. We also performed OGTT at discharge and 3 months after discharge.

**Results:**

The prevalence of disturbances in glucose metabolism (as assessed by OGTT) at 3 months was significantly lower than at discharge (29% *vs*. 48%, p = 0.0001). Disturbances in glucose metabolism were not confirmed in 63% of patients with impaired glucose tolerance and in 36% of patients with diabetes mellitus diagnosed during the acute phase of AMI. Age >77 years, glucose ≥12.06 mmol/l at 120 minutes during OGTT before discharge and mean blood glucose level on day 2 >7.5 mmol/l were identified as independent predictors of disturbances in glucose metabolism at the 3-month follow-up.

**Conclusions:**

Disturbances in glucose metabolism observed in patients with a first AMI are predominantly transient. Elderly age, high plasma glucose concentration at 120 minutes during OGTT at discharge and elevated mean blood glucose level on day 2 were associated with sustained disturbances in glucose metabolism.

## Introduction

European guidelines on diabetes, pre-diabetes, and cardiovascular diseases [1] recommend the performance of an oral glucose tolerance test (OGTT) in patients with established cardiovascular disease. Furthermore, the guidelines on management of acute myocardial infarction in patients presenting with persistent ST-segment elevation [2] specify that an OGTT should be performed before or shortly after hospital discharge. However, data supporting such suggestions are inconclusive.

Norhammar *el al. *[3] stated that disturbances in glucose metabolism diagnosed on the basis of fasting glucose and OGGT in patients with acute myocardial infarction (AMI) are previously undiagnosed pathologies related to impairment in β-cell function rather than stress-induced hyperglycaemia. In the GAMI study [4] the same research group confirmed that OGTT performed in AMI patients at hospital discharge gives reliable information about long-term glucometabolic state. Conversely, in another Scandinavian study the number of AMI subjects with disturbances in glucose metabolism on OGTT fell from 46.9% when measured in-hospital to 24.9% at three months [5].

The aim of our study was to analyze whether disturbances in glucose metabolism diagnosed before hospital discharge in AMI patients represent a latent pre-existing condition or rather temporary finding. Additionally, we planned the investigation of pre-selected glycaemic control parameters in terms of their utility as predictors of long-term glucometabolic state in patients with first acute myocardial infarction.

## Methods

### Patients and study design

We conducted a prospective observational cohort study in consecutive patients with no prior history of diabetes who were hospitalized between November 2005 and December 2007 for a first episode of ST-segment-elevation acute myocardial infarction. AMI was diagnosed according to international recommendations [6]. Exclusion criteria were: ongoing treatment with catecholamines, glucocorticoids or immunosuppressive agents; severe congestive heart failure (NYHA III and IV); plasma creatinine concentration >2 mg/dl; malignant disease or clinically significant infection.

Among 261 eligible patients we excluded 61 patients from the further analysis due to lack of any assessed diabetic control parameters (n = 27), unwillingness to participate in the study (n = 20), failure to attend the follow-up appointment (n = 8) and presence of exclusion criteria (n = 6). Therefore, the final sample comprised 200 patients.

The following parameters were evaluated: admission glycaemia in venous blood plasma, glycated hemoglobin (HbA_1c_) on day 1, fasting and post-prandial (2 hours after 3 main meals) capillary blood glucose on day 1 and day 2, as well as 75 g OGTT immediately before discharge and at 3 months after discharge. Mean blood glucose concentrations on days 1 and 2 were obtained from 4 capillary blood samples.

Glucose concentrations were determined using a hexokinase method (Glucose kit, Abbott, Lake County, USA), and an immunoturbidymetric method (Multigent™Hb_A1c_, Seradyn Inc. Indianapolis, USA) was used to measure HbA_1c_.

We divided patients into 3 groups on the basis of their plasma glucose concentration at 120 minutes of OGTT: normal glucose tolerance (NGT) defined as plasma glucose concentration <7.8 mmol/l; impaired glucose tolerance (IGT) defined as plasma glucose concentration ≥7.8 mmol/l but <11.1 mmol/l); new onset diabetes (DM) defined as plasma glucose concentration ≥11.1 mmol/l. Disturbances in glucose metabolism were defined as IGT or DM.

During hospitalization we also assessed: total cholesterol (TC), high density lipoproteins (HDL) and triglycerides (TG) - enzymatic kits by Abbott (Lake County, USA). LDL concentration was calculated according to the Friedewald formula.

The study was certified by the local ethics committee. All participants have given voluntary written informed consent for inclusion in the study.

### Statistical analysis

Use of the Shapiro-Wilk test demonstrated that the investigated variables were not normally distributed. Parameters are presented as arithmetic mean (M) ± standard deviation (SD) for quantitative data or as percentage distribution (%) for qualitative attributes. We applied suitable non-parametric tests (the Mann-Whitney unpaired rank sum test, the Kruskal-Wallis unpaired rank sum test, and the Wilcoxon matched-paired rank sum test) for comparisons of quantitative dependent and independent variables. For evaluation of correlations between quantitative variables we used the Spearman's correlation coefficient and the test of significance for this coefficient. Qualitative data were analyzed with the χ^2 ^test (including Yates' correction when indicated) or the McNamara test for unrelated and related qualitative variables respectively.

We used logistic regression and discriminant analysis to identify the best predictors for the occurrence of disturbances in glucose metabolism at 3 months after discharge. For specified laboratory tests distinguished by high discriminative power, receiver operating characteristic (ROC) curves were drawn in order to estimate their diagnostic yield. The cut-off value for statistical significance was defined as p < 0.05. Statistical analysis was performed using STATISTICA 8.0 (StatSoft, Tulsa, United States).

## Results

Patients with a pathological result of OGTT 3 months after AMI were older than patients with NGT. A direct comparison between patients with NGT and those with DM at 3 months revealed higher values of admission glycaemia, HbA_1c, _mean glycaemia on day 1 and day 2 of hospitalization in diabetic patients. The IGT group was distinguished from the NTG and DM groups by significantly higher mean glycaemia on day 2 and markedly lower HbA_1c _level, respectively (Table 1). We found no differences among the groups in the location of AMI, treatment course or levels of myocardial necrosis markers (data not presented).

**Table 1:** Baseline characteristics of study groups with normal glucose tolerance, impaired glucose tolerance and diabetes mellitus as assessed with oral glucose tolerance test at the 3-month follow-up (n = 200).

	Overall study population N = 200	Normal glucose tolerance N = 142	Impaired glucose tolerance N = 48	Diabetes mellitus N = 10	p
Age (years)	56.5 ± 8.7	54.9 ± 8.3	60.6 ± 8.1	59.8 ± 8.7	0.00012*

Male n (%)	155 (77.5)	112 (78.9)	35 (83.3)	8 (80)	NS

Body mass index (kg/m^2^)	27.0 ± 4.0	26.3 ± 4.1	26.7 ± 4.1	28.9 ± 3.1	NS

Admission glycemia (mmol/l)	7.5 ± 1.6	7.3 ± 1.5	7.8 ± 1.7	8.8 ± 1.9	0.016**

HbA_1c _(%)	6.0 ± 0.7	6.0 ± 0.7	5.9 ± 0.6	7.1 ± 0.6	0.0001** 0.00006***

Mean blood glycemia on day 1 (mmol/l)	6.5 ± 1.0	6.4 ± 0.9	6.8 ± 0.9	7.8 ± 1.6	0.03**

Mean blood glycemia on day 2 (mmol/l)	6.8 ± 1.0	6.6 ± 0.8	7.3 ± 1.3	7.7 ± 0.7	0.002** 0.0003*

Total cholesterol (mmol/l)	5.8 ± 1.1	5.8 ± 1.1	5.9 ± 1.4	6.0 ± 1.1	NS

LDL cholesterol (mmol/l)	3.9 ± 1.0	3.9 ± 0.9	3.8 ± 1.2	3.8 ± 0.9	NS

HDL cholesterol (mmol/l)	1.4 ± 0.3	1.4 ± 0.3	1.4 ± 0.3	1.3 ± 0.2	NS

Triglycerides (mmol/l)	1.3 ± 1.1	1.2 ± 0.8	1.5 ± 1.6	2.0 ± 1.6	NS

Creatinine (μmol/l)	84.8 ± 15.4	84.8 ± 14.7	83.6 ± 16.8	91.5 ± 18.5	NS

Hypertension (%)	36.5	31.7	47.9	50	NS
Current smoker (%)	70.5	71.8	64.6	80	NS
Family history of premature coronary artery disease (%)	24.5	26.1	22.9	10	NS

* normal glucose tolerance *vs*. impaired glucose tolerance, ** normal glucose tolerance *vs*. diabetes mellitus, *** impaired glucose tolerance *vs*. diabetes mellitus

HbA_1c _- glycated hemoglobin.

**Figure 1:**
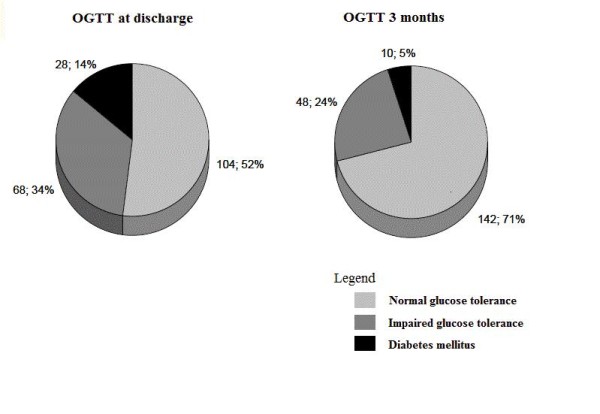
**Prevalence of disturbances in glucose metabolism according to OGTT performed before hospital discharge and after 3 months**. OGTT - oral glucose tolerance test.

As presented on Figure 1 disturbances in glucose metabolism assessed by OGTT were significantly more frequent before hospital discharge than at 3-months (48 *vs*. 29%; p = 0.0001). At 3 months there were no disturbances in glucose metabolism in 63 and 36% of patients diagnosed during the acute phase of AMI with IGT and DM, respectively. In the IGT group disturbances in glucose metabolism persisted for at least 3 months in 37% of patients, while the incidence of disturbances in glucose metabolism after 3 months in the DM group was 64% (Table 2).

**Table 2:** Results of OGTT immediately before discharge and 3 months after discharge (n = 200).

OGTT immediately before discharge n (%)		OGTT 3 months after discharge n (%)		
		
		normal glucose tolerance	impaired glucose tolerance	diabetes mellitus
normal glucose tolerance	104 (52%) *	89 (86%)	15 (14%)	-

impaired glucose tolerance	68 (34%) **	43 (63%)	23 (34%)	2 (3%)

diabetes mellitus	28 (14%) ***	10 (36%)	10 (36%)	8 (28%)

total	200 (100%)	142 (71%) *	48 (24%) **	10 (5%) ***

p		0.0004 *	0.0001 **	0.0001 ***

p - for comparison of patients in the particular subgroup immediately before discharge and 3 months after discharge

OGTT - oral glucose tolerance test.

Patients with an abnormal result of OGTT performed before discharge but a normal test at 3 months had a significantly higher mean blood glucose on days 1 and 2 as well as triglyceride concentration when compared to patients with a normal response to both tests (Table 3).

**Table 3:** Comparison between patients with an abnormal result of OGTT performed before discharge but a normal test at 3 months and patients with a normal response to both tests.

	IGT (n = 43) or DM before discharge (n = 10) and normal OGTT at 3 months	NGT before discharge and at 3 months (n = 89)	p
Age (years)	56.0 ± 8.3	54.2 ± 8.4	NS

Male n (%)	39 (73.6)	73 (82.0)	NS

Body mass index (kg/m^2^)	26.6 ± 5.2	26.1 ± 3.4	NS

Admission glycemia (mmol/l)	7.4 ± 1.5	7.2 ± 1.5	NS

HbA_1c _(%)	6.2 ± 0.9	5.9 ± 0.5	NS

Mean blood glycemia on day 1 (mmol/l)	6.7 ± 1.0	6.2 ± 0.9	< 0.009

Mean blood glycemia on day 2 (mmol/l)	6.8 ± 0.7	6.4 ± 0.8	< 0.006

Total cholesterol (mmol/l)	5.9 ± 1.0	5.7 ± 1.1	NS

LDL cholesterol (mmol/l)	3.8 ± 0.9	3.9 ± 0.9	NS

HDL cholesterol (mmol/l)	1.4 ± 0.3	1.4 ± 0.3	NS

Triglycerides (mmol/l)	1.5 ± 1.1	1.0 ± 0.5	< 0.002

Creatinine (μmol/l)	85.7 ± 16.4	84.2 ± 13.6	NS

Hypertension (%)	17 (32.1)	28 (31.5)	NS

Current smoker (%)	39 (73.6)	63 (70.8)	NS

Family history of premature coronary artery disease (%)	16 (30.2)	21 (23.6)	NS

Activity of CK-MB on admission (U/l)	52.0 ± 83.2	45.9 ± 58.5	NS

Concentration of troponin I on admission (ng/mL)	1.9 ± 7.0	2.4 ± 7.5	NS

LVEF at discharge (%)	43.4 ± 6.3	44.8 ± 6.5	NS

Heart failure symptoms at discharge (%)	3 (5.7)	3 (3.4)	NS

CK-MB - isoenzyme MB of creatine kinase; DM - diabetes mellitus; HbA_1c _- glycated hemoglobin; IGT - impaired glucose tolerance; LVEF - left ventricular ejection fraction; NTG - normal glucose tolerance; OGTT - oral glucose tolerance test.

**Figure 2:**
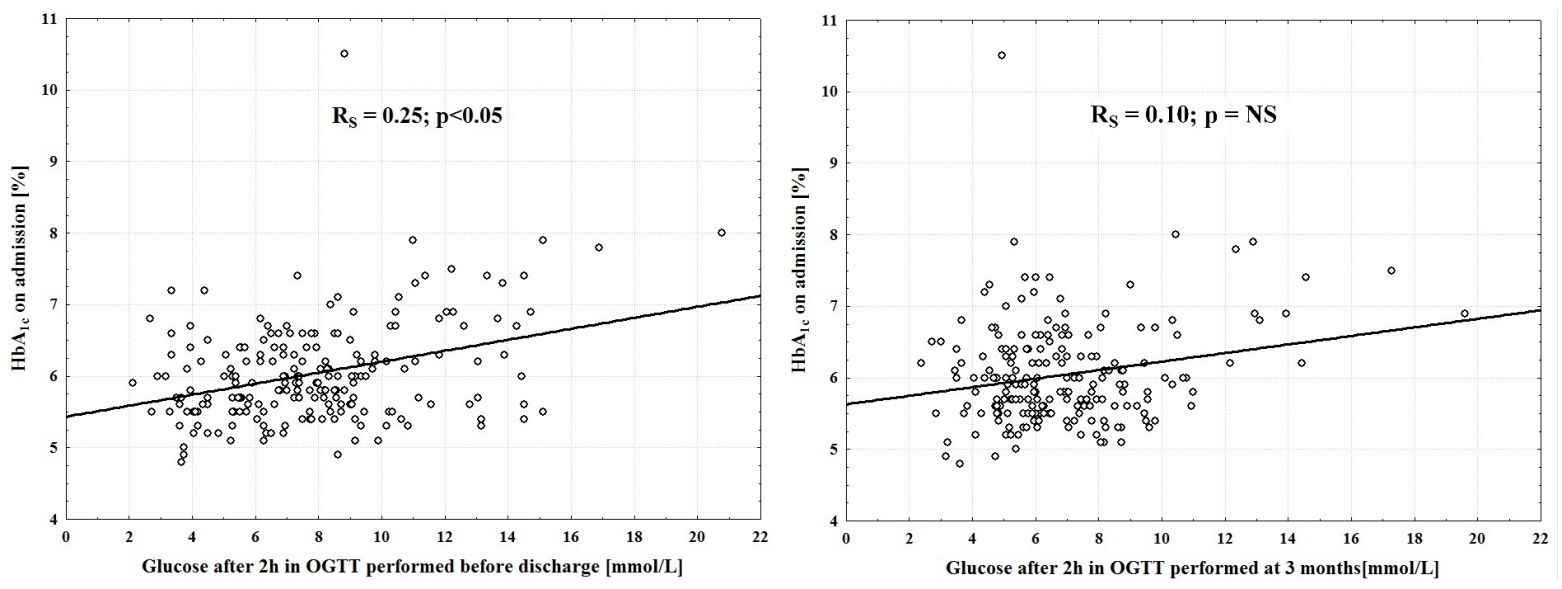
**Relations between HbA_1c _assessed on admission and plasma glucose after 2 hours in OGTT performed before discharge and at 3 months, respectively**. HbA_1c _- glycated hemoglobin; OGTT - oral glucose tolerance test; R_S _- the Spearman's rank correlation coefficient.

HbA_1c _on admission only weakly correlated with plasma glucose after 2 hours in OGTT performed before discharge (Figure 2). We observed no impact of HbA_1c _assessed on admission on the result of OGTT at 3 months.

All variables displaying significant differences among the subgroups and gender were included into computational models in order to define independent predictors for the presence of disturbances in glucose metabolism 3 months after myocardial infarction. In logistic regression analysis the value of goodness-of fit χ2 test p < 0.00001 was calculated. The following variables were found as independent predictors of disturbances in glucose metabolism at 3-month follow-up: age (OR 0.94; 95% CI 0.88-0.99; p < 0.05), glucose concentration at 120 min of OGTT immediately before discharge (OR 0.75; 95% CI 0.628-0.899; p = 0.002) and mean glycaemia on day 2 (OR 0.44; 95% CI 0.22-0.81; p = 0.009). The discriminative analysis confirmed the above parameters such as elderly age (p = 0.009), high glucose level at 120 minutes of OGTT before hospital discharge (p < 0.0003) and elevated mean glycaemia on day 2 (p < 0.05) as powerful predictors of disturbances in glucose metabolism at the 3-month follow-up. The ROC curve analysis performed in order to assess diagnostic accuracy for the prediction of sustained glucometabolic disturbances revealed area under the curve (AUC) = 0.716 with cut-off value of 12.056 mmol/l for glucose level at 120 minutes of OGTT before hospital discharge (p = 0.044), AUC = 0.696 with cut-off value of 77 years for age (p = 0.04) as well as AUC = 0.727 with cut-off value of 7.514 mmol/l for mean glycaemia on day 2 (p = 0.045), respectively.

## Discussion

Hyperglycemia diagnosed in the peri-infarct period is clinically highly relevant [7-12]. The prevalence of disturbances in glucose metabolism in acute myocardial infarction in our study was 48% which is comparable to other published data. The Euro Heart Survey [13] reported the combined incidence of new-onset IGT and DM to be 58%, while in The China Heart Survey [14] it was 45% (IGT 24% and DM 21%).

On the other hand, our data indicate significantly lower rates of DM and IGT at the 3-month follow-up when compared to the peridischarge period. Therefore, recommendation in the current guidelines on the performance of OGTT before or shortly after the hospital discharge might not be applicable to all patients. Similarly, Knudsen *et al. *[5] found that a substantial number of patients with abnormal glucose tolerance in the peri-infarct period had normal glucose tolerance 3 months after their AMI. However, in the cited study the OGTT test was performed very early i.e. during the first day after admission. The divergence between both studies and the data obtained by Swedish researchers [3,4], who reported a high reproductability of OGTT performed in the acute phase of AMI and after 3 months, may derive, at least partly, from age differences between study cohorts. Age difference derives from the fact that only patients with first episode of myocardial infarction were enrolled into our study. Younger patients, with well preserved function of the pancreatic β cells, may develop disturbances in glucose metabolism only in the setting of insulin resistance induced by metabolic stress. When stress is eliminated, the insulin secretion becomes adequate to maintain normal glucose levels. In contrast, in older populations the gradual degradation of the pancreatic β-cells is responsible for disturbances in glucose metabolism development, which often remains clinically silent and therefore may be identified only when concomitant ailments (e.g. AMI) occur.

Our doubts concerning the studies conducted by the Swedish group also result from their other analysis [15] where they presented a poor reproducibility of OGTT. As much as 52% of patients of the NGT group at the time of AMI developed either IGT or DM at the 3-month follow-up. The coherence in diagnosis of disturbances in glucose metabolism at discharge and in the 3-month follow-up accounted for only 49% of cases. On the other hand, we found that 3 months after myocardial infarction IGT appeared in only 14% of patients originally classified as NGT compared with 86% of subjects who remained free from disturbances in glucose metabolism. Moreover, none of the NGT patients developed DM in the course of the 3-month follow-up.

AMI survivors have considerably higher yearly morbidity rates of diabetes and impaired fasting glucose than individuals who have not had an AMI [16]. Therefore, it seems clinically relevant to identify simple to interpret and feasible criteria that might predict the persistence of disturbances in glucose metabolism after an AMI. According to our data as well as other previously published works [3,15,17,18] admission glucose level has insufficient prognostic value. We found that the strongest predictor of disturbed glucose metabolism at 3 months was glucose concentration >12.06 mmol/l at 120 minutes in the pre-discharge OGTT. Similarly, Norhammar *et al. *[3] and Tenerz *et al. *[15] found a close correlation between peak glucose concentration in OGTT performed immediately before discharge and after 3 months. However, authors of the first [3] study were unable to establish a cut-off value for the risk of persistent disturbances in glucose metabolism while in the latter [15] glucose was only measured at 60 minutes in the OGTT. This fact is in contrast to the statement of World Health Organization [19] that considers the concentration of glucose at 120 minutes in OGTT as an essential criterion for diagnosing different forms of disturbances in glucose metabolism. HbA_1c _assessment on admission to hospital as a predictor for previously undiagnosed disturbances in glucose metabolism has also been verified in several studies [3,5,15,17,18]. Nevertheless, their results are inconclusive. Furthermore, we established mean blood glucose on day 2 as an independent risk factor for disturbances in glucose metabolism at 3 month follow-up. To the best of our knowledge these parameters have not yet been validated in other studies.

### Study limitations

We cannot excluded the possibility that reduction in daily calorie intake or decrease in daily carbohydrate consumption within the first days of myocardial infarction might produce false negative results in OGTT tests performed in this period. Limited reproducibility of an OGTT should be also taken into account. Nevertheless this test remains the most valuable tool for early recognition of individuals with diabetes or at increased risk for diabetes and heart disease [20].

## Conclusions

Disturbances in glucose metabolism observed in patients with a first AMI are predominantly transient. Elderly age, high blood glucose concentration at 120 minutes during OGTT immediately before hospital discharge and elevated mean blood glucose concentration on day 2 are associated with sustained disturbances in glucose metabolism.

## Competing interests

The authors declare that they have no competing interests.

## Authors' contributions

AB, RJ, and JK were responsible for the design conception of the study. AB, MK, and JK interpreted the data and wrote the manuscript. PM and AS analysed the data. TF, JG, AS and ZG enrolled patients and performed follow-up visits. IS collected the data, contributed to the interpretation of results, and supervised the study. AK and MB critically revised manuscript. All authors have read and approved the final manuscript.
